# Advances in modern mental chronometry

**DOI:** 10.3389/fnhum.2015.00256

**Published:** 2015-05-06

**Authors:** José M. Medina, Willy Wong, José A. Díaz, Hans Colonius

**Affiliations:** ^1^Departamento de Óptica, Facultad de Ciencias, Universidad de GranadaGranada, Spain; ^2^Department of Electrical and Computer Engineering, Institute of Biomaterials and Biomedical Engineering, University of TorontoToronto, ON, Canada; ^3^Department für Psychologie, Carl von Ossietzky Universität OldenburgOldenburg, Germany

**Keywords:** mental chronometry, reaction time, timing and time perception, sensory perception, cognition, human performance, stochastic processes, decision making

Mental chronometry encompasses all aspects of time processing in the nervous system and constitutes a standard tool in many disciplines including theoretical and experimental psychology and human neuroscience. Mental chronometry has represented a fundamental approach to elucidate the time course of many cognitive phenomena and their underlying neural circuits over more than a century. Nowadays, mental chronometry continues evolving and expanding our knowledge, and our understanding of the temporal organization of the brain in combination with different neuroscience techniques and advanced methods in mathematical analysis. In research on mental chronometry, human reaction/responses times (RT) play a central role. Together with RTs, other topics in mental chronometry include vocal, manual and saccadic latencies, subjective time, psychological time, interval timing, time perception, internal clock, time production, time representation, time discrimination, time illusion, temporal summation, temporal integration, temporal judgment, redundant signals effect, perceptual, decision and motor time, etc. It is worth noting that there have been well over 37,000 full-length journal papers published in the last decade on a variety of topics related to simple and choice RTs, etc. This amounts to approximately 3800 papers per year, or roughly 10 papers per day (source: PubMed, similarly Thomson Reuters Web of Science). There are comprehensive reviews that deal extensively with the history of mental chronometry, experimental methods and paradigms, stochastic models, etc. as well as its relationship to other psychological and physiological variables, neuroscience methods and clinical applications (Laming, 1968; Posner, 1978, 2005; Welford and Brebner, 1980; Townsend and Ashby, 1983; Luce, 1986; Meyer et al., 1988; Robbins and Brown, 1990; Schall, 2001; Mauk and Buonomano, 2004; Smith and Ratcliff, 2004; Jensen, 2006; Gold and Shadlen, 2007; Linden, 2007; Grondin, 2010; Merchant et al., 2013; Allman et al., 2014).

The aim of this research topic is to provide an overview of the state of the art in this field—its relevance, recent findings, current challenges, perspectives and future directions. Thus, as a result, a collection of 14 original research and opinion papers from different experts have been gathered together in a single volume. They outline a selection of unsolved problems and topics in mental chronometry mainly within the context of the human visual system as well as the auditory system. One of the unsolved problems is the functional role of power laws in RT variability and in the study of timing. Power laws are ubiquitous in many complex systems, and their experimental validity and theoretical support represent a fundamental aspect in many disciplines, such as in biology, physics, finance, etc. In this theme issue, the papers of Ihlen (2014), Medina et al. (2014), Rigoli et al. (2014) and Shouval et al. (2014) address different aspects of power laws, namely, multifractal analysis on RT series; an information theoretic basis of RT power law scaling; Fourier-based power law correlations (“1/*f* noise”) in a tapping task and its comparison with other physiological processes (e.g., heartbeat intervals); and a log-power law model of the firing rate of neurons in interval timing.

A second unsolved problem involves RT-based methods and research into RT distributions. RT distributions are typically positively skewed and often exhibit long right-tails in the time-domain. A long-standing issue deals with the shape of RT distributions, their intrinsic stochastic latency mechanisms and neural basis. Sequential-sampling models are a common approach widely used in human RTs and simple decision making (Smith and Ratcliff, 2004). Diederich and Oswald present a RT sequential-sampling model for multiple stimulus features based on an Ornstein–Uhlenbeck diffusion process (Diederich and Oswald, 2014). In a different type of analysis, the work of Harris et al. introduces an alternative approach to examine very long RTs in the rate-domain (i.e., 1/RT). These authors investigate the shape of choice RT distributions and sequential correlations using autoregressive techniques (Harris et al., 2014). In general, RT distributions exhibit faster RTs under summation/facilitation tasks when two or more redundant signals are available as compared with a single signal or sensory modality (e.g., binocular vs. monocular vision), usually called redundant signals effect. The work of Lentz et al. examines binaural vs. monaural hearing performance under noise masking tasks using modeling techniques based on the concept of workload capacity and different processing mechanisms (e.g., serial vs. parallel, etc.) and stopping rules (Lentz et al., 2014). Within the same redundant signals paradigm, Zehetleitner et al. study bimodal (audio-visual) facilitation effects using sequential-sampling models (Zehetleitner et al., 2015).

Regarding the human vision system, the work of Wegener et al. examines the visual attention mechanisms using colored stimuli (random dot patterns), and they have presented a novel three-step model of attention to predict the corresponding RT distributions (Wegener et al., 2014). The work of Murd et al. exemplifies the used RTs in conjunction with visual evoked potentials in the detection of visual colored stimuli (Murd et al., 2014). There are also studies focusing on the auditory system, including the work of Nakajima et al. that investigates the foundations of time perception using a time illusion based on an overestimation of a second time interval preceded by a first time interval or time-shrinking effect (Nakajima et al., 2014). Mitsudo et al. present recorded magnetoencephalogram signals in tasks that require to judge temporal gaps in tones and have discussed their implications in the organization of the auditory cortex (Mitsudo et al., 2014). Within the same time perception paradigm, Mioni et al. show a detailed review on temporal dysfunctions in traumatic brain injury patients (Mioni et al., 2014). The present theme issue also includes the work of García-Pérez who introduces a unified model to analyze different psychophysical tasks in time perception and estimation of the psychometric function (García-Pérez, 2014).

We hope this research topic will provide a useful framework and an up-to-date set of papers for further discussion on mental chronometry within the human brain.

## Conflict of interest statement

The authors declare that the research was conducted in the absence of any commercial or financial relationships that could be construed as a potential conflict of interest.

## References

[B1] Allman M. J. Teki S. Griffiths T. D. Meck W. H. (2014). Properties of the internal clock: first- and second-order principles of subjective time. Annu. Rev. Psychol. 65, 743–777. 10.1146/annurev-psych-010213-115117 24050187

[B2] Diederich A. Oswald P. (2014). Sequential sampling model for multiattribute choice alternatives with random attention time and processing order. Front. Hum. Neurosci. 8:697. 10.3389/fnhum.2014.00697 25249963 PMC4158981

[B3] García-Pérez M. A. (2014). Does time ever fly or slow down? The difficult interpretation of psychophysical data on time perception. Front. Hum. Neurosci. 8:415. 10.3389/fnhum.2014.00415 24959133 PMC4051264

[B4] Gold J. I. Shadlen M. N. (2007). The neural basis of decision making. Annu. Rev. Neurosci. 30, 535–574. 10.1146/annurev.neuro.29.051605.113038 17600525

[B5] Grondin S. (2010). Timing and time perception: a review of recent behavioral and neuroscience findings and theoretical directions. Atten. Percept. Psychophys. 72, 561–582. 10.3758/APP.72.3.561 20348562

[B6] Harris C. Waddington J. Biscione V. Manzi S. (2014). Manual choice reaction times in the rate-domain. Front. Hum. Neurosci. 8:418. 10.3389/fnhum.2014.00418 24959134 PMC4051275

[B7] Ihlen E. A. F. (2014). Multifractal analyses of human response time: potential pitfalls in the interpretation of results. Front. Hum. Neurosci. 8:523. 10.3389/fnhum.2014.00523 25100972 PMC4104308

[B8] Jensen A. R. (2006). Clocking the Mind: Mental Chronometer Individual Differences. Amsterdam: Elsevier.

[B9] Laming D. (1968). Information Theory of Choice-reaction Times. San Diego, CA: Academic Press.

[B10] Lentz J. He Y. Townsend J. T. (2014). A new perspective on binaural integration using response time methodology: super capacity revealed in conditions of binaural masking release. Front. Hum. Neurosci. 8:641. 10.3389/fnhum.2014.00641 25202254 PMC4141468

[B11] Linden D. E. (2007). What, when, where in the brain? Exploring mental chronometry with brain imaging and electrophysiology. Rev. Neurosci. 18, 159–171. 10.1515/REVNEURO.2007.18.2.159 17593878

[B12] Luce R. D. (1986). Response Times. New York, NY: Oxford University Press.

[B13] Mauk M. D. Buonomano D. V. (2004). The neural basis of temporal processing. Annu. Rev. Neurosci. 27, 307–340. 10.1146/annurev.neuro.27.070203.144247 15217335

[B14] Medina J. M. Díaz J. A. Norwich K. (2014). A theory of power laws in human reaction times: insights from an information-processing approach. Front. Hum. Neurosci. 8:621. 10.3389/fnhum.2014.00621 25161618 PMC4129233

[B15] Merchant H. Harrington D. L. Meck W. H. (2013). Neural basis of the perception and estimation of time. Annu. Rev. Neurosci. 36, 313–336. 10.1146/annurev-neuro-062012-170349 23725000

[B16] Meyer D. E. Osman A. M. Irwin D. E. Yantis S. (1988). Modern mental chronometry. Biol. Psychol. 26, 3–67. 10.1016/0301-0511(88)90013-0 3061480

[B17] Mioni G. Grondin S. Stablum F. (2014). Temporal dysfunction in traumatic brain injury patients: primary or secondary impairment? Front. Hum. Neurosci. 8:269. 10.3389/fnhum.2014.00269 24817847 PMC4012215

[B18] Mitsudo T. Hironaga N. Mori S. (2014). Cortical activity associated with the detection of temporal gaps in tones: a magnetoencephalography study. Front. Hum. Neurosci. 8:763. 10.3389/fnhum.2014.00763 25346672 PMC4191557

[B19] Murd C. Kreegipuu K. Kuldkepp N. Raidvee A. Tamm M. Allik J. (2014). Visual evoked potentials to colour change of a moving bar. Front. Hum. Neurosci. 8:19. 10.3389/fnhum.2014.00019 24478683 PMC3900876

[B20] Nakajima Y. Hasuo E. Yamashita M. Haraguchi Y. (2014). Overestimation of the second time interval replaces time-shrinking when the difference between two adjacent time intervals increases. Front. Hum. Neurosci. 8:281. 10.3389/fnhum.2014.00281 24860471 PMC4030194

[B21] Posner M. I. (1978). Chronometric Explorations of Mind. Oxford: Lawrence Erlbaum.

[B22] Posner M. I. (2005). Timing the brain: mental chronometry as a tool in neuroscience. PLoS Biol. 3:e51. 10.1371/journal.pbio.0030051 15719059 PMC548951

[B23] Rigoli L. M. Holman D. Spivey M. Kello C. (2014). Spectral convergence in tapping and physiological fluctuations: coupling and independence of 1/f noise in the central and autonomic nervous systems. Front. Hum. Neurosci. 8:713. 10.3389/fnhum.2014.00713 25309389 PMC4160925

[B24] Robbins T. W. Brown V. J. (1990). The role of the striatum in the mental chronometry of action: a theoretical review. Rev. Neurosci. 2, 181–214. 10.1515/REVNEURO.1990.2.4.181 21561254

[B25] Schall J. D. (2001). Neural basis of deciding, choosing and acting. Nat. Rev. Neurosci. 2, 33–42. 10.1038/35049054 11253357

[B26] Shouval H. Z. Hussain Shuler M. G. Agarwal A. Gavornik J. P. (2014). What does scalar timing tell us about neural dynamics? Front. Hum. Neurosci. 8:438. 10.3389/fnhum.2014.00438 24994976 PMC4063330

[B27] Smith P. L. Ratcliff R. (2004). Psychology and neurobiology of simple decisions. Trends Neurosci. 27, 161–168. 10.1016/j.tins.2004.01.006 15036882

[B28] Townsend J. T. Ashby F. G. (1983). The Stochastic Modeling of Elementary Psychological Processes. Cambridge: Cambridge University Press.

[B29] Wegener D. Galashan F. O. Aurich M. K. Kreiter A. K. (2014). Attentional spreading to task-irrelevant object features: experimental support and a 3-step model of attention for object-based selection and feature-based processing modulation. Front. Hum. Neurosci. 8:414 10.3389/fnhum.2014.00414 PMC4051263 24959132

[B30] Welford A. T. Brebner J. M. T. (1980). Reaction Times. New York, NY: Academic Press.

[B31] Zehetleitner M. Ratko-Dehnert E. Mueller H. J. (2015). Modeling violations of the race model inequality in bimodal paradigms: co-activation from decision and non-decision components. Front. Hum. Neurosci. 9:119 10.3389/fnhum.2015.00119 PMC4353255 25805987

